# Numerical and Experimental Study on the Molten Pool Behavior and Magnetic Properties of Nano-Crystalline Alloy Ribbon Prepared by PlanarFlow Casting

**DOI:** 10.3390/ma19081510

**Published:** 2026-04-09

**Authors:** Lijun Li, Hongxin Ji, Jianliang Sun, Deren Li, Baisong Li, Jintao Yao

**Affiliations:** 1 Advanced Technology and Materials Co., Ltd., China Iron and Steel Research Institute Group, Beijing 100081, China; lilijun@atmcn.com (L.L.); libaisong@atmcn.com (B.L.); yaojintao@atmcn.com (J.Y.); 2 National Engineering Research Center for Equipment and Technology of Cold Strip Rolling, Yanshan University, Qinhuangdao 066004, China; jihongxin@stumail.ysu.edu.cn; 3 School of Electrical Engineering, Beijing Jiaotong University, Beijing 100044, China; derenli@bjtu.edu.cn

**Keywords:** planar-flow casting, nanocrystalline, soft magnetic material, molten pool, ribbon thickness, numerical simulation

## Abstract

A 2D multiphase-flow coupling simulation model for preparing nanocrystalline ribbons using planar-flow casting (PFC) with a cooling roller was established. The influence of roller speed on molten pool characteristics, cooling-roller heat transfer, and ribbon thickness was analyzed. The effect of ribbon thickness on the total loss and permeability of the magnetic cores was investigated. The results indicate that the molten pool size decreased as the roller speed increased. At t = 5 ms, the maximum heat-transfer coefficient of the roller surface increased from 2.09 × 10^6^ W·m^−2^·K^−1^ at 15 m/s to 2.6 × 10^6^ W·m^−2^·K^−1^ at 24 m/s. The ribbon thickness decreased from 39.96 μm to 20.02 μm (a 49.9% reduction) as the roller speed increased from 18 m/s to 30 m/s. The total loss of the nanocrystalline magnetic cores increased with ribbon thickness, whereas their permeability increased as ribbon thickness decreased. At 100 kHz, the nanocrystalline magnetic core made of 10–12 μm ribbons exhibited a high permeability of 59,507.

## 1. Introduction

Fe-based amorphous–nanocrystalline ribbons possess high-saturation magnetic induction, high effective permeability, and low coercivity, which can be fabricated into various magnetic cores [1,2,3]. With the advancement of electronic information technology towards higher frequencies and greater integration, there is an urgent need to prepare magnetic cores that can exhibit excellent soft magnetic properties at higher operating frequencies [4].

Scholars have extensively investigated the preparation of amorphous–nanocrystalline magnetic cores with excellent performance [5,6]. Geng et al. [7] found that varying the longitudinal magnetic-field intensity of annealing significantly affects the magnetic properties of Fe_73_._7_Si_15_._3_Cu_1_Nb_3_B_7_ nanocrystalline magnetic cores. Han et al. [8] optimized the soft magnetic properties of FeSiBNbCu alloy strips by adjusting the B/Si ratio. In addition, ribbon thickness is a key factor affecting the soft magnetic properties of the magnetic core [9]. Investigating the influence of ribbon thickness on core soft magnetic properties is significant for extending the application of these cores in fields like new energy vehicles and power electronics.

**Figure 1:**
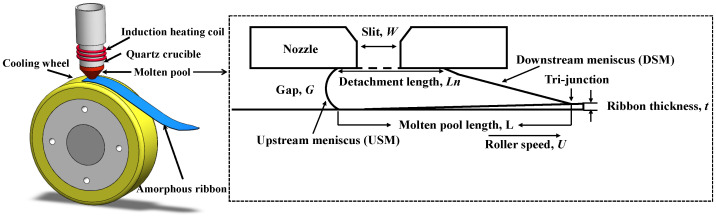
Schematic diagram of the PFC process (not to scale) [10].

Planar-flow casting (PFC) is one of the most important rapid solidification techniques for manufacturing amorphous–nanocrystalline ribbons [10]. During the PFC process, the molten alloy is sprayed onto a high-speed rotating cooling copper roller under overpressure. A molten pool is formed between the nozzle and the cooling roller, which is constrained by surface tension. The melt is pulled out of the molten pool by the rapidly rotating cooling rollers and quickly solidifies (10^5^–10^7^ K/s) into ribbons (Figure 1).

The PFC is a non-equilibrium solidification process characterized by high temperature, pressure, and speed [11]. This process involves multiphase flow, complex heat, and mass transfer, coupled with the interaction of multiple physical fields. Numerical simulation can observe fluid dynamic behavior in the molten pool over a very short period of time. It is an effective method to investigate molten pool behavior. Liu et al. [12] developed a two-dimensional (2D), multiphase flow-coupling model for preparing amorphous ribbons using PFC. The flow and heat-transfer behaviors of the molten pool were studied. Tawfeeq et al. [13] analyzed the formation process of the molten pool during the preparation of Fe-Si-B and Ni-B ribbons via PFC using a 2D simulation model. Nevertheless, few studies have employed numerical simulation to elucidate the key parameters for fabricating thinner ribbons using PFC.

Therefore, research remains limited on the influence of ribbon thickness on the soft magnetic properties of nanocrystalline cores, as well as on the effects of PFC parameters on ribbon thickness. In this paper, a 2D simulation model was established that couples the volume of fluid (VOF) method for multiphase flow with the PFC process to simulate the preparation of nanocrystalline alloy ribbons, considering the cooling roller. The melt flow and heat-transfer behavior were theoretically analyzed. The influence of the key parameter of roller speed on molten pool shape and ribbon thickness was discussed. Magnetic cores were fabricated using Fe_73.5_Cu_1_Nb_3_Si_13.5_B_9_ nanocrystalline ribbons (ribbon thicknesses are 10–12 μm, 12–14 μm, and 14–16 μm). The magnetic properties of nanocrystalline magnetic cores with different ribbon thicknesses were studied. This work provides a reference for the fabrication of magnetic cores by applying higher frequencies.

## 2. Numerical Modeling

### 2.1. The Simulation Model for Planar-Flow Casting

**Figure 2:**
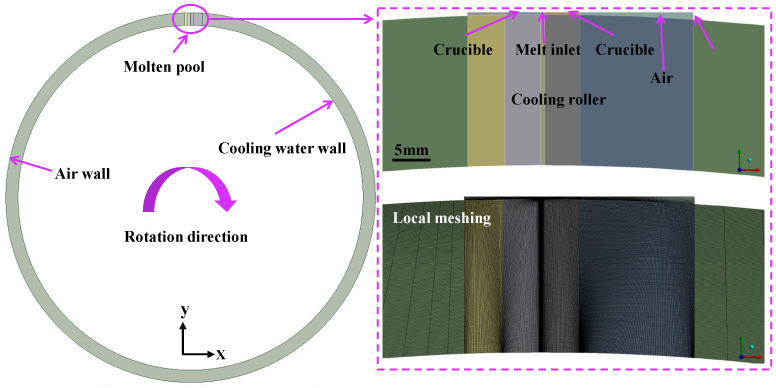
The 2D simulation modeling for the preparation of amorphous ribbons and local meshing.

In this paper, a 2D simulation model for the preparation of amorphous ribbons using PFC with a cooling roller was established (Figure 2). The simulation analysis was conducted using ANSYS Fluent 2021 R1, with a time step of 1 × 10^−6^ s. The roller thickness is 20 mm, the roller–nozzle gap is 0.3 mm, and the nozzle slit width is 0.4 mm. The entire zone was divided into quadrilateral grids, and the grids in key areas were refined. Eventually, 69,885 nodes and 69,494 elements were generated, with the minimum orthogonal quality reaching 0.9, indicating that the grid division results meet the calculation requirements. A VOF model with a geometric reconstruction scheme was used to track the air–melt interface. The transient, pressure-based coupling solver was set up, and the splitting of operators (PISO) pressure–velocity coupling method was used. The momentum and energy equations were then solved using a first-order upwind scheme across the entire zone.

### 2.2. Fundamental Assumptions

(1) Compared to the ribbon thickness, its width and length can be considered as infinite. Therefore, the model is simplified to a 2D analysis of flow and heat transfer.
(2) All other parameters are set as constants and do not change with temperature, except for melt viscosity.
(3) The molten material and air in the molten pool are considered to be incompressible Newtonian fluids, and the flow pattern is assumed to be laminar.
(4) Due to the extremely fast cooling rate during the PFC process, the release of latent heat during the solidification of the melt can be neglected during the extremely short solidification process.

### 2.3. Mathematical Model

The following equations were employed in the simulation [14]:

Continuity equation:

(1) $$\frac{\partial \rho }{\partial t}+\frac{\partial (\rho u_{i})}{\partial x_{i}}=0$$

Momentum equation:

(2) $$\rho (\frac{\partial (u_{i})}{\partial t}+\frac{\partial (u_{i}u_{j})}{\partial x_{j}})=\frac{\partial }{\partial x_{j}}\left[\mu \left[\frac{\partial u_{i}}{\partial x_{j}}+\frac{\partial u_{j}}{\partial x_{i}}\right]\right]-\frac{\partial P}{\partial x_{i}}+\rho g+f$$

where ρ refers to the density (kg/m^3^); t is time (s); $u_{i}$ and $u_{j}$ indicate the velocity components (m/s); $x_{i}$ and $x_{j}$ denote the directional components; μ is the viscosity (Pa·s); P denotes the pressure (Pa); f is the surface tension source phase; and g is the gravitational acceleration (m/s^2^).

Energy equations:

(3) $$\rho (\frac{\partial T}{\partial t}+\frac{\partial \left(C_{p}u_{i}T\right)}{\partial x_{i}})=\frac{\partial }{\partial x_{i}}\left(K\frac{\partial T}{\partial x_{i}}\right)$$

(4) $$\frac{\partial }{\partial_{t}}\left(\rho_{cr}C_{p,cr}T\right)+\frac{\partial }{\partial x_{i}}\left(\rho_{cr}C_{p,cr}{\left(\omega R\right)}_{i}T\right)=\frac{\partial }{\partial x_{i}}\left(K_{cr}\frac{\partial T}{\partial x_{i}}\right)$$

where T indicates temperature (K); K represents the thermal conductivity (W/(m·K)); $C_{p}$ is the constant pressure specific heat (J/kg^−1^K^−1^); cr refers to the cooling roller; ω signifies the rotation speed of the roller (rad/s); and R is the radius of the cooling roller (m).

The VOF model is used to track the evolution of the air–melt two-phase interface, with the volume fraction *F* determined by the following equations.

(5) $$\frac{\partial F}{\partial t}+u_{i}\frac{\partial F}{\partial x_{i}}=0$$

(6) $$F=\left\{\begin{array}{l} 0,The\;entire\;calculation\;domain\;is\;filled\;with\;air\\ 0<F<1,air+melt\\ 1.0,The\;entire\;calculation\;domain\;is\;filled\;with\;melt \end{array}\right.$$

The thermophysical parameters of air and melt appearing in the transport equation depend on the value of F within each cell, which is computed using the equations below.

(7) $$f=\sigma \frac{\rho K\nabla F}{\frac{1}{2}\left(\rho_{a}+\rho_{m}\right)}$$

(8) $$k=-\nabla \cdot \frac{\nabla F}{\left|\nabla F\right|}$$

(9) $$\begin{array}{l} \rho =\rho_{a}F+\rho_{m}\left(1-F\right)\\ \mu =\mu_{a}F+\mu_{m}\left(1-F\right)\\ C_{p}=C_{p,a}F+C_{p,m}\left(1-F\right)\\ k=k_{a}F=k_{m}\left(1-F\right) \end{array}$$

where ‘a’ stands for air and ‘m’ stands for amorphous materials.

The expression for the viscosity of the amorphous alloy is as follows [12]:

(10) $$\mu =0.10\times \exp\left(-3.6528+734.1/\left(T-674\right)\right)$$

The isothermal contour of the melt at $T_{g}$ = 873 K is taken as the solid–liquid separation boundary. In the present work, a tri-junction (J*) is defined as the location where the downstream meniscus meets the $T_{g}$. The ribbon thickness is defined as the vertical gap between J* and the roller surface. Table 1 lists the key process parameters of the PFC method.

The convective heat-transfer coefficient is determined from the heat flux value, as given by the equation below.

(11) $$H=q/(T_{wall}-T_{\infty })$$

where H is the convective heat-transfer coefficient (W·m^−2^·K^−1^); q indicates the heat flux (W·m^−2^); $T_{wall}$ denotes the roller surface temperature (K); and $T_{\infty }$ is the initial temperature of the copper roller and surrounding air (K).

## 3. Experiments

**Figure 3:**
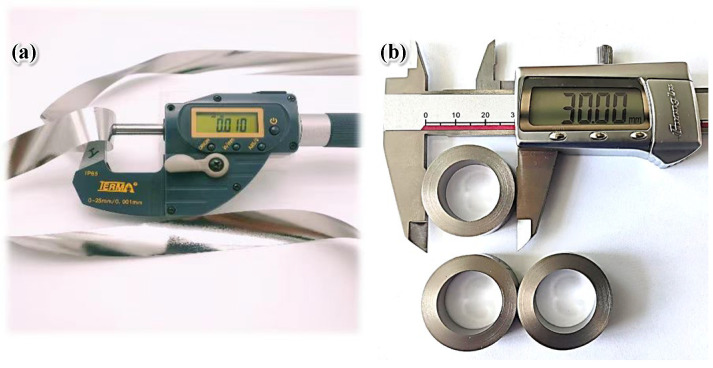
(**a**) Fe_73.5_Cu_1_Nb_3_Si_13.5_B_9_ amorphous ribbon; (**b**) nanocrystalline magnetic cores.

The material used in this paper was an Fe_73.5_Cu_1_Nb_3_Si_13.5_B_9_ alloy. The amorphous ribbons with thicknesses of 10–12 μm (Figure 3a), 12–14 μm, and 14–16 μm were prepared by PFC. The ribbon was cut to a width of 10 mm. Crystallization heat treatment was carried out on three thicknesses of nanocrystalline ribbons. The temperature was increased rapidly to 480 °C at 8 °C/min and held for 30 min. Then, the temperature was slowly increased to 560 °C at 2 °C/min and held for 60 min to nanocrystallize the amorphous alloy. The final annealing temperature of 560 °C was selected to prevent the precipitation of the hard magnetic Fe_3_B phase. After annealing, α-Fe nanocrystals were uniformly dispersed within the amorphous matrix. The ribbons were cooled to 200 °C under argon protection, then naturally cooled to room temperature. As illustrated in Figure 3b, the ribbons after crystallization heat treatment were wound by the winding machine to form magnetic cores (outer diameter 30 mm, inner diameter 20 mm, and height 10 mm). The magnetic cores were subjected to transverse magnetic-field annealing. The temperature was increased at 5 °C/min to 400 °C and held for 60 min. The magnetic cores were cooled in the furnace to 200 °C, with air cooling to room temperature. The single-turn inductance of the magnetic core was measured at different frequencies at a test voltage of 0.5 V using a precision impedance analyzer (Agilent E4294A), and the magnetic permeability was derived from these measurements. The total loss of the magnetic cores under different conditions was tested using the IWATSU B-H Analyzer. Each test was repeated three times to get reproducible results.

## 4. Results and Discussion

### 4.1. The Characteristics of the Molten Pool Under Different Roller Speeds

**Figure 4:**
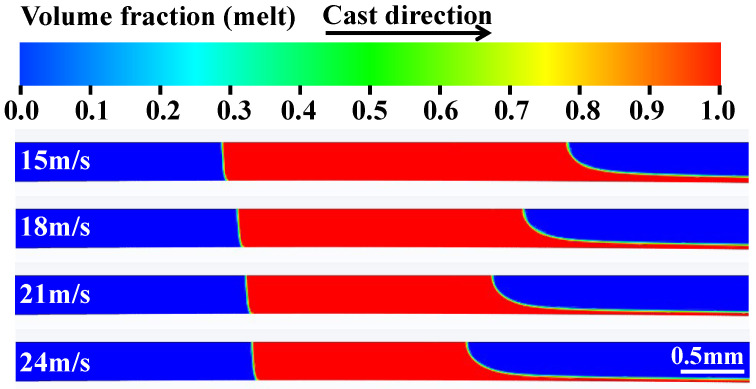
The molten pool shapes under different roller speeds.

At $T_{e}$ = 1623 K and V = 1.6 m/s, the size of the molten pool decreased as the roller speed increased, as illustrated in Figure 4. As the U was increased from 15 m/s to 24 m/s, the detachment length (Ln) decreased from 2.68 mm to 1.65 mm, and the molten pool length (L) decreased from 5.41 mm to 3.47 mm. Ln decreased by 38.4%, and L decreased by 35.9%. The offset of the upstream meniscus (USM) to the center of the nozzle slit is very small, but that of the downstream meniscus (DSM) is very large. At high roller speeds, the melt is drawn out of the molten pool more quickly by the roller surface. Therefore, DSM moves further towards the center of the nozzle slit [15].

### 4.2. Temperature Field Distribution During the PFC Process

**Figure 5:**
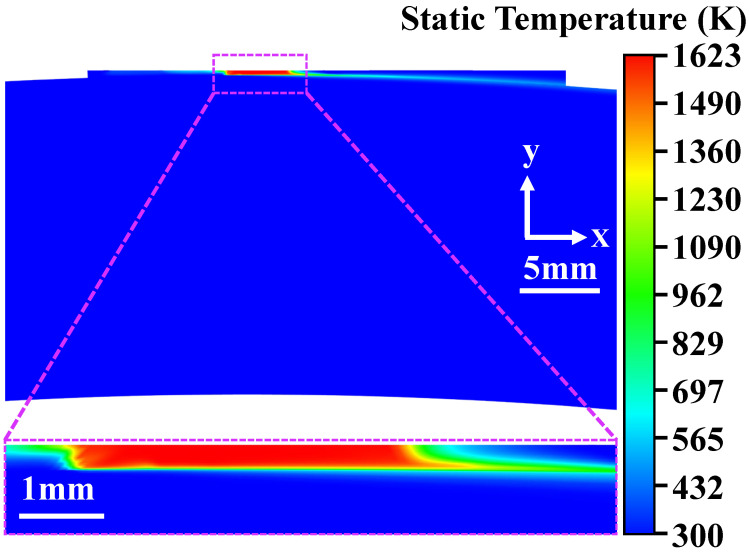
Temperature distribution in the copper roller and molten pool.

The casting ribbon parameters are T = 1623 K, V = 1.6 m/s, and U = 18 m/s. As shown in Figure 5, at t = 5 ms, the temperature in the molten pool is uniform and is the same as the melt injection temperature. Layered isothermal lines existed around the molten pool and the cooling roller surface, resulting in a temperature gradient. The molten pool size is so small that radiation heat transfer can be neglected. Most of the heat was carried away by the rotating cooling copper roller, and the temperature decreased as the rotational arc length increased. The temperature inside the cooling roller gradually rises due to the influence of heat conduction.

### 4.3. The Impact of Varying Roller Speeds on Important Parameters

**Figure 6:**
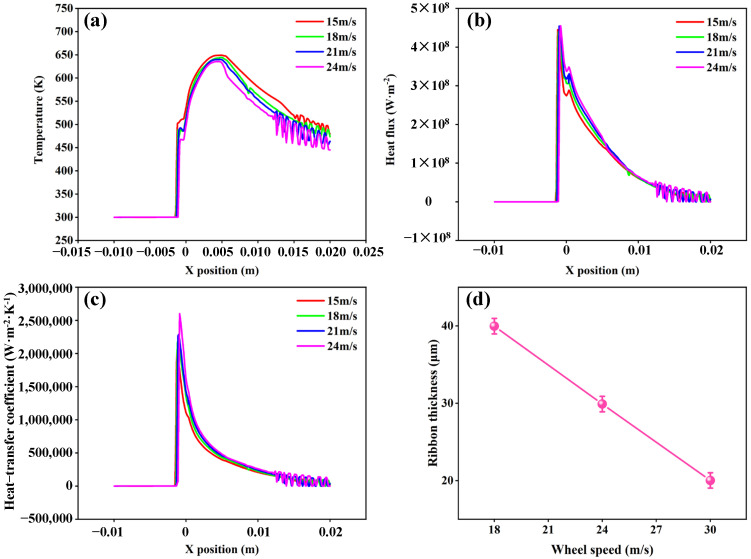
The simulation results under different roller speeds: (**a**) roller surface temperature; (**b**) roller surface heat flux; (**c**) roller surface convective heat-transfer coefficient; (**d**) ribbon thickness.

In the downstream area of the molten pool (X > 0 mm), the roller surface temperature exhibited a decreasing trend, as illustrated in Figure 6a. For example, at U = 21 m/s, the temperature decreased from 640 K (x = 0.0048 mm) to 463.6 K (x = 0.02 mm). With the increase in roller speed from 15 m/s to 24 m/s, the maximum surface temperature decreased from 649.2 K to 635.6 K. The heat flux on the roller surface dropped sharply at first, then decreased gradually to a steady state, as shown in Figure 6b. This is due to the heat transfer between the molten pool and the cooling roller. The heat flux on the roller surface increased with roller speed. The heat-transfer coefficient of the roller surface is an important parameter in the PFC process. The convective heat-transfer coefficient was not constant between the molten pool and roller surface, but decreased exponentially as x increased. At high roller speeds, the convective heat-transfer coefficient at the interface is also greater. The maximum heat-transfer coefficient increased from 2.09 × 10^6^ W·m^−2^·K^−1^ at 15 m/s to 2.6 × 10^6^ W·m^−2^·K^−1^ at 24 m/s, as shown in Figure 6c. It can be seen that at higher roller speeds, the time for the melt to solidify was shorter and the cooling rate was greater. As the roller speed increased from 18 m/s to 30 m/s, the ribbon thickness decreased from 39.96 μm to 20.02 μm, a reduction of 49.9% (Figure 6d).

### 4.4. Magnetic Properties of Fe_73.5_Cu_1_Nb_3_Si_13.5_B_9_ Nanocrystalline Cores with Different Thicknesses

**Figure 7:**
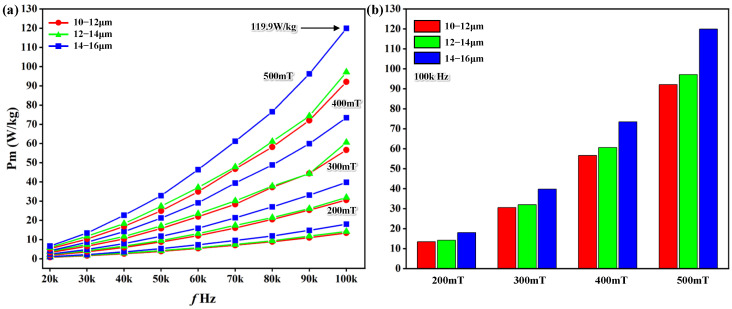
The specific total loss of different-ribbon-thickness Fe_73.5_Cu_1_Nb_3_Si_13.5_B_9_ nanocrystalline cores: (**a**) under different maximum applied flux density; (**b**) under the 100 kHz frequency condition.

The total specific loss of nanocrystalline magnetic cores of different thicknesses is shown in Figure 7. The total specific loss increased as the maximum applied flux density increased. For example, the maximum magnetic flux density of a magnetic core with 14–16 μm ribbon thickness increased from 200 mT to 500 mT, and the total loss increased from 18.01 W/kg to 119.9 W/kg, as illustrated in Figure 7a. At the same maximum applied flux density (200 mT), the core loss increased as the frequency increased. For example, for a 12–14 µm ribbon-thickness nanocrystalline magnetic core, as the frequency increased from 20 kHz to 100 kHz, the total loss increased from 0.92 W/kg to 14.16 W/kg (Figure 7a). At a fixed frequency, greater ribbon thickness led to higher total loss. The ribbon thickness is 10–12 μm at 100 kHz (500 mT). The total specific loss of the magnetic core is lowest at 92.11 W/kg, which is 23.2% lower than the 14–16 μm ribbon-thickness magnetic core (119.9 W/kg), as shown in Figure 7b.

**Figure 8:**
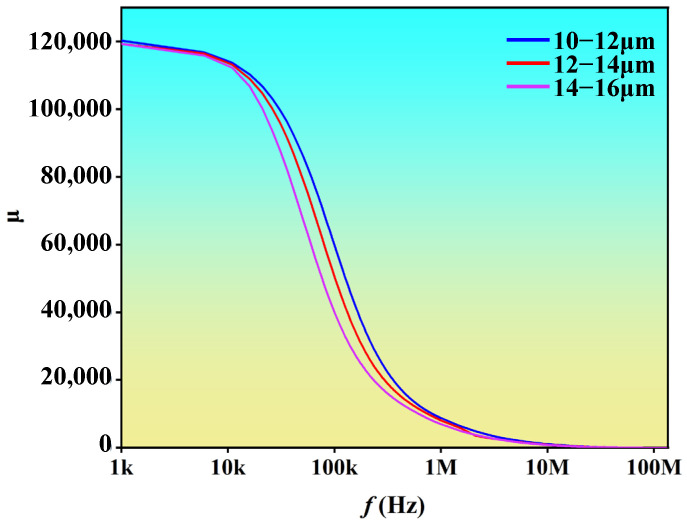
Permeability—frequency curves for nanocrystalline magnetic cores of different ribbon thicknesses.

Figure 8 shows the relationship between the permeability and frequency of nano-crystalline magnetic cores with different ribbon thicknesses. The magnetic cores exhibit relatively high permeability within the frequency range of 10 kHz to 100 kHz. The permeability decreased sharply at first and then slowly as the frequency increased. Since hysteresis loss ($P_{h}\propto f$) and eddy current loss ($P_{e}\propto f^{2}$) both increase with frequency, the total core loss exhibits a rapid upward trend as frequency increases. At high frequencies, the permeability of the cores decreases rapidly with increasing frequency, which is closely related to the magnetization dynamics. At 100 kHz, reducing the ribbon thickness from 14–16 μm to 10–12 μm increased the magnetic core permeability from 39,749 to 59,507, a rise of 49.7%. Nanocrystalline magnetic cores fabricated from thinner ribbons exhibit higher permeability at medium- and high frequencies.

## 5. Conclusions

(1) As the roller speed was increased from 15 m/s to 24 m/s, the Ln decreased from 2.68 mm to 1.65 mm, and the L decreased from 5.41 mm to 3.47 mm. Ln decreased by 38.4%, and L decreased by 35.9%. Most of the heat was carried away by the rotating cooling roller, and the temperature decreased as the rotational arc length increased.
(2) At t = 5 ms, the roller surface temperature increased with increasing roller speed. The maximum heat-transfer coefficient increased from 2.09 × 10^6^ W·m^−2^·K^−1^ at 15 m/s to 2.6 × 10^6^ W·m^−2^·K^−1^ at 24 m/s. As the roller speed increased from 18 m/s to 30 m/s, the ribbon thickness decreased from 39.96 μm to 20.02 μm, a reduction of 49.9%.
(3) The total loss of the nanocrystalline magnetic core increased with increasing ribbon thickness. At 500 mT and 100 kHz, the 10–12 μm magnetic core exhibited a minimum loss of 92.11 W/kg. Permeability was found to increase as the ribbon thickness decreased. At 100 kHz, the 10–12 μm magnetic core reached a high permeability of 59,507.

## Figures and Tables

**Table 1:** The PFC process parameters employed in the simulation model [10].

Parameters	Values
Roller radius (R)	0.3 m
Slit width of nozzle (W)	0.4 mm
Initial temperature (*T*_0_)	300 K
Ejection temperature of melt (*T_e_*)	1623 K
Gap distance (G)	0.3 mm
Melt ejection speed (V)	1.6 m/s
Surface tension of alloy (σ)	1.2 N/m

## Data Availability

The original contributions presented in this study are included in the article. Further inquiries can be directed to the corresponding author.
